# Directional emission of plastic luminescent films using photonic crystals fabricated by soft-X-ray interference lithography and reactive ion etching

**DOI:** 10.1038/s41598-018-27593-w

**Published:** 2018-06-18

**Authors:** Qiang Wu, Bo Liu, Zhichao Zhu, Mu Gu, Hong Chen, Chaofan Xue, Jun Zhao, Yanqing Wu, Renzhong Tai, Xiaoping Ouyang

**Affiliations:** 1 Shanghai Key Laboratory of Special Artificial Microstructure Materials and Technology, School of Physics Science and Engineering, Tongji University, Shanghai, 200092 P. R. China; 2 Shanghai Institute of Applied Physics, Chinese Academy of Sciences, Shanghai Synchrotron Radiation Facility, Shanghai, 201800 P. R. China; 3 State Key Laboratory of Intense Pulsed Radiation Simulation and Effect, Northwest Institute of Nuclear Technology, Xi’an, 710024 P. R. China

## Abstract

In this report, a novel method to prepare photonic crystals based on the combination of soft-X-ray interference lithography (XIL) and reactive ion etching (RIE) with a bi-layer photoresist system was developed. XIL can be utilized to prepare periodic structures with high efficiency but the depth of etch is limited due to the strong absorption of photoresist for soft-X-ray. Based on the pattern prepared by XIL, RIE can be utilized to further etch a second layer of photoresist, so that one can obtain a large depth of etch. Controlling the dispersion relation of the prepared photonic crystals, strongly directional emission of plastic luminescent films was demonstrated. A wavelength-integrated enhancement of 2.64-folds enhancement in the range of 420 to 440 nm in the normal direction was obtained. Guided-mode resonance and Fabry-Perot resonance could be the critical factors to control the directional emission. Devices based on directional emission films have a variety of applications in such as detectors, optical communication and display screens.

## Introduction

Plastic luminescent films are extensively investigated for a wide range of applications including organic light-emitting diodes in electroluminescence devices^1–5^, luminescent solar concentrators for photovoltaic^6–8^ and scintillators in nuclear radiation detection systems^9,10^. However, for the applications of planar luminescent films, a majority of light is trapped by the total internal reflection due to high refractive indices forming in-plane guided-wave modes which cannot be efficiently collected by photodetectors. Additionally, the small portion of light which can emit from the films usually follows a Lambertian angular profile without specific directionality^11^, giving rise to a low detection efficiency in practical detection systems.

In recent years, with the development of nanotechnology, nanostructures have attracted increasing interest for their potential applications in a variety of fields including optical modulators^12–14^, bio-sensors^15–18^, photovoltaic devices^19,20^, and light emission devices^21–23^. Nanostructures can achieve efficient control of light based on the modulation of optical dispersion relations or optical mode density of states^24,25^. For example, biologically inspired moth-eye-like nanostructures have been utilized to enhance the light output of Lu_2_SiO_5_:Ce luminescent film^26^. Photonic crystals forming with an array of monolayer polystyrene microspheres have been applied to improve the light extraction efficiency^27,28^. Two-dimensional photonic crystals can also be used to control the directionality of light emission based on the theory of guided-mode resonances^29–32^.

The most frequently used fabrication methods for photonic structures with nanoscale include direct-writing lithography, self-assembly, nanoimprint and interference lithography. However, each method has its own limitation. For instance, electron beam lithography is a direct writing technique which can obtain high resolution nanostructures, but it is unfavorable for fabricating large area nanostructures due to its high fabrication cost. Self-assembly method enables large-area fabrication, but the periodic array is not perfect in the whole region and limited in several tens of micrometers. Nanoimprint technology can prepare large area nanostructures with low cost, but is not suitable for the preparation of fragile samples. Recently, soft X-ray interference lithography (XIL) technique is a rapidly developing technique based on coherent radiation obtained from undulators at synchrotron radiation facility^33–35^. Due to the ability to fabricate periodic structures with high efficiency, XIL is advantageous to photonic applications^36,37^. Combined with large-area stitching technique, the area of periodic nanostructures up to several square millimeters could be readily fabricated within tens of minutes^38^. In addition, the advantages of its noncontact lithography method and no requirement for substrate conductivity make it possible for extensive manufacturing fields.

In our previous work, XIL has been utilized to prepare photonic crystal structures on the surface of an emitting crystal, enhancing the light extraction efficiency^39^. However, the obtained height/depth of the photonic crystal structures was only several tens of nanometers due to the strong absorption of the conventional photoresist, which restricts the application of XIL in the fields where sufficient height/depth and high-aspect ratio are required.

In this report, in order to obtain photonic crystals with a sufficient height, we propose a method based on bi-layer photoresist pattern fabricated by the combination of XIL and reactive ion etching (RIE). The prepared photonic crystals can be used to control the directional emission of luminescent films, which has potential applications in photoelectric devices, light emitting devices and nuclear radiation detections. The photonic crystals with an array of nanopillars were prepared on the surface of a quartz substrate. A layer of plastic luminescent film was spinning coated onto the photonic crystal layer forming a structured luminescent layer which will exhibit controllable emission character. The prepared plastic luminescent film is a kind of plastic scintillator which can play an important role in the field of radiation detection. Devices based on the directional emission with improved collection efficiency of scintillation light have a variety of applications in such as scintillation detectors and scintillation screen.

**Figure 1:**
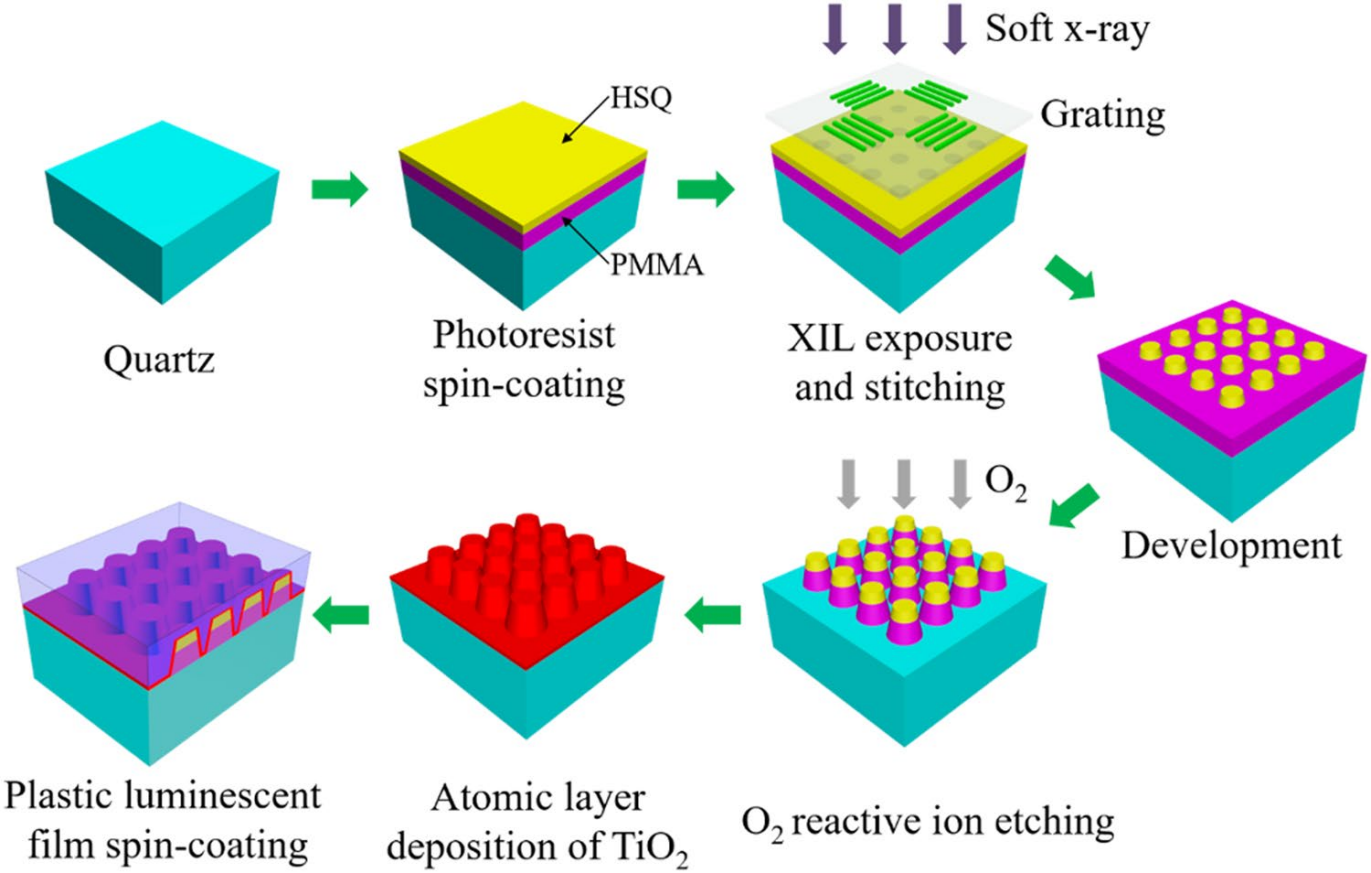
Schematic illustration of the fabrication process of the two-dimensional photonic crystal structures coated with plastic luminescent films.

Figure 1 is the schematic diagram of the fabrication process of a two-dimensional photonic crystal structures on the surface of a quartz substrate. The fabrication processes include the pattern process of hydrogen silsesquioxane (HSQ) resist by XIL and the subsequent pattern process of poly-methyl-methacrylate (PMMA) resist by RIE. HSQ is a commonly used resist for XIL preparation^40,41^. To protect the prepared pattern and increase the contrast of the refractive index, the conformal layer of high refractive index TiO_2_ (n = 2.8) was deposited on the surface of the nanopillars using an atomic layer deposition (ALD) technique.

**Figure 2:**
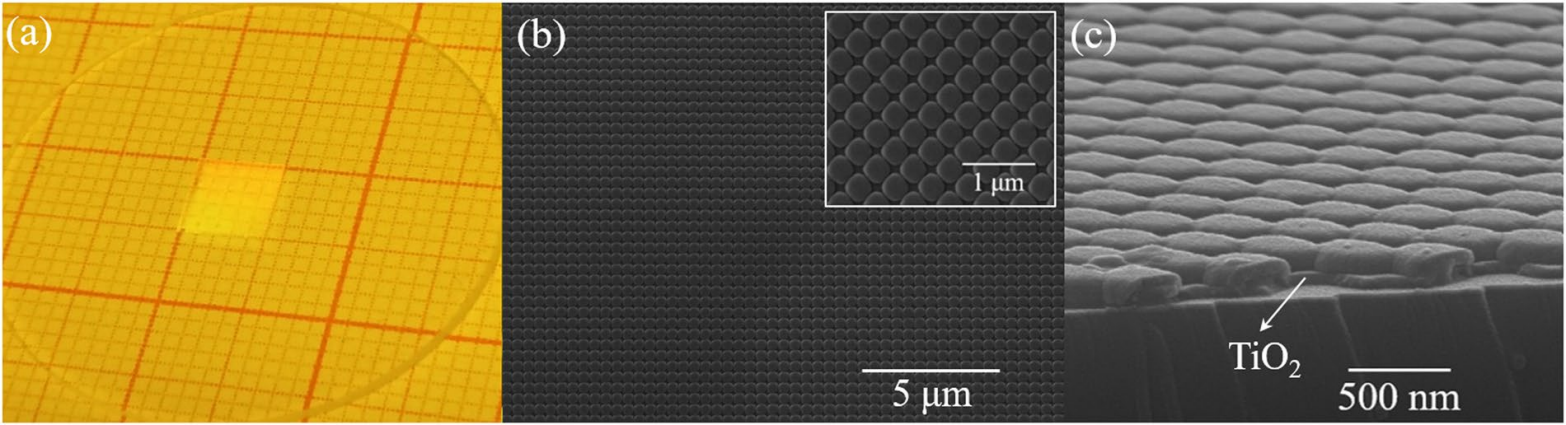
**(a)** The picture of the prepared photonic crystals on a quartz substrate. SEM images of the photonic crystal structures for **(b)** the top view and **(c)** the oblique view.

**Figure 3:**
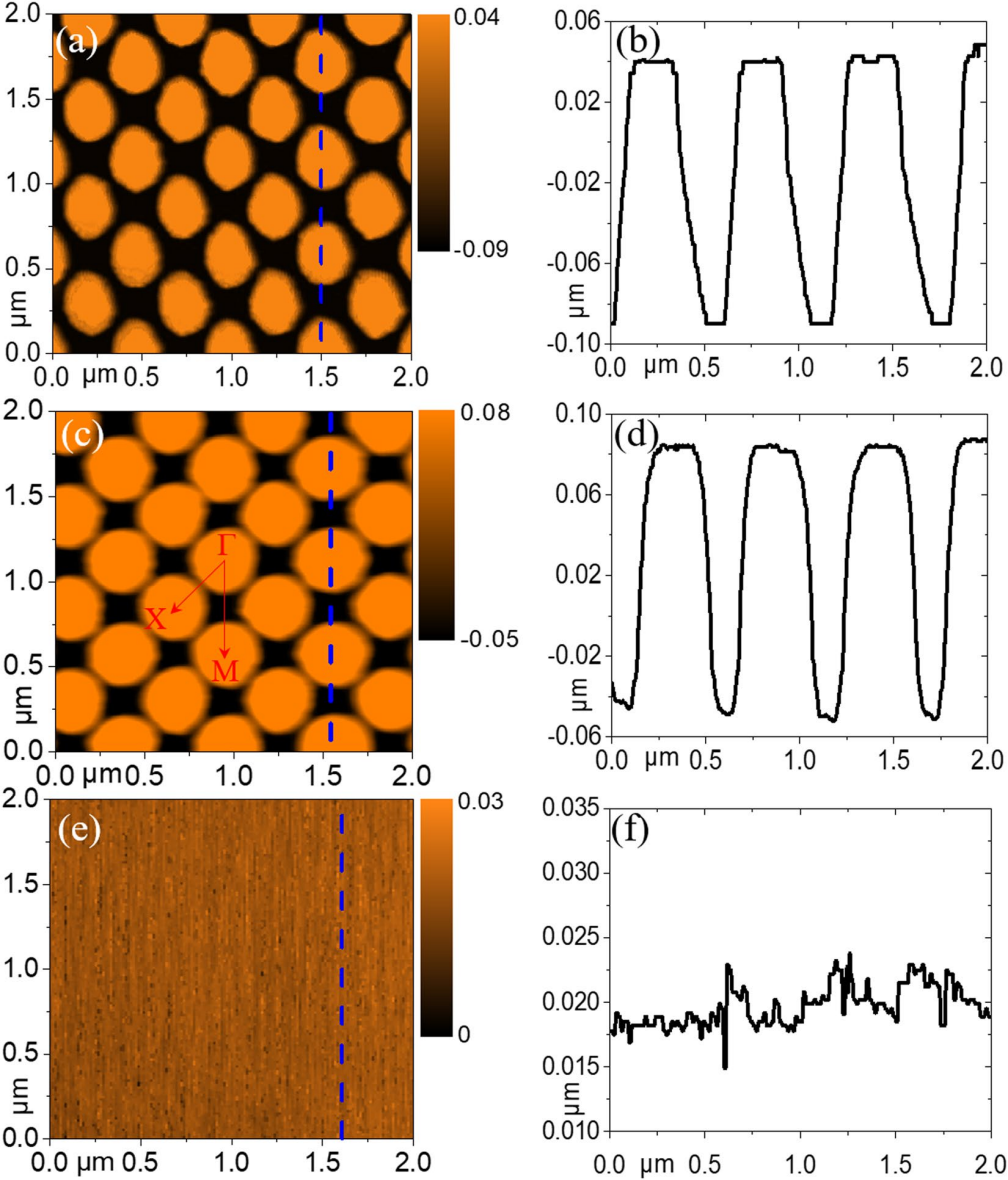
AFM images of the photonic crystal structures before **(a)** and after **(c)** the deposition of conformal TiO_2_ layer with the line scan profiles **(b)** and **(d)** along the dashed lines in **(a)** and **(c)**, respectively. AFM image of the morphology after coated with luminescent film **(e)** with the line scan profile along the blue dashed line **(f)**.

The picture of the prepared two-dimensional photonic crystals on quartz substrate is shown in Fig. 2(a), indicating that there are no apparent defects or particulate contamination, and the structural region exhibits good diffraction patterns. Figure 2(b,c) show scanning electron microscopy (SEM) images of the photonic crystals for the top view and the oblique view, respectively, exhibiting periodicity and homogeneous aspect ratio. In addition, the TiO_2_ conformal layer is deposited on the surface of nanopillars uniformly. The height and the period of the photonic crystals before and after the deposition of conformal TiO_2_ layer were checked by atomic force microscope (AFM), shown in Fig. 3(a–d). The orientations of ΓM and ΓX are defined in Fig. 3(c). It is found that the photonic crystals belong to a square lattice with the lattice constant of 400 nm. The final obtained nanopillars were composed of two parts and the total height of the individual nanopillars was about 130 nm. The deposition rate of TiO_2_ in the side wall is related to the spacing between nanopillars. The larger the column spacing is, the more TiO_2_ is deposited. So the prepared patterns in Fig. 3(a) are elliptical and they become circular in Fig. 3(c) after depositing TiO_2_ layer. The plastic luminescent film with a thickness of 450 nm was covered on the prepared photonic crystals by spin-coating method, obtaining a structured luminescent film. Figure 3(e) shows the final surface morphology of luminescent film checked by AFM and the line scan profile along the blue dashed line is presented in Fig. 3(f), indicating that the surface roughness of the plastic luminescent film is less than 15 nm. There are narrow gaps of several micrometers in the process of stitching and the nanopatterns close to the edges are not very uniform. The defects occupy a relatively small area so that the stitching errors do not seriously affect our experimental and theoretical analysis. The photonic crystal structure only covered a small part of the quartz substrate. The part of plastic luminescent film where there is no photonic crystal structure was regarded as a reference sample which maintains a planar structure. In addition, the theoretical model of photonic crystal structures established later is based on the measured surface morphology.

**Figure 4:**
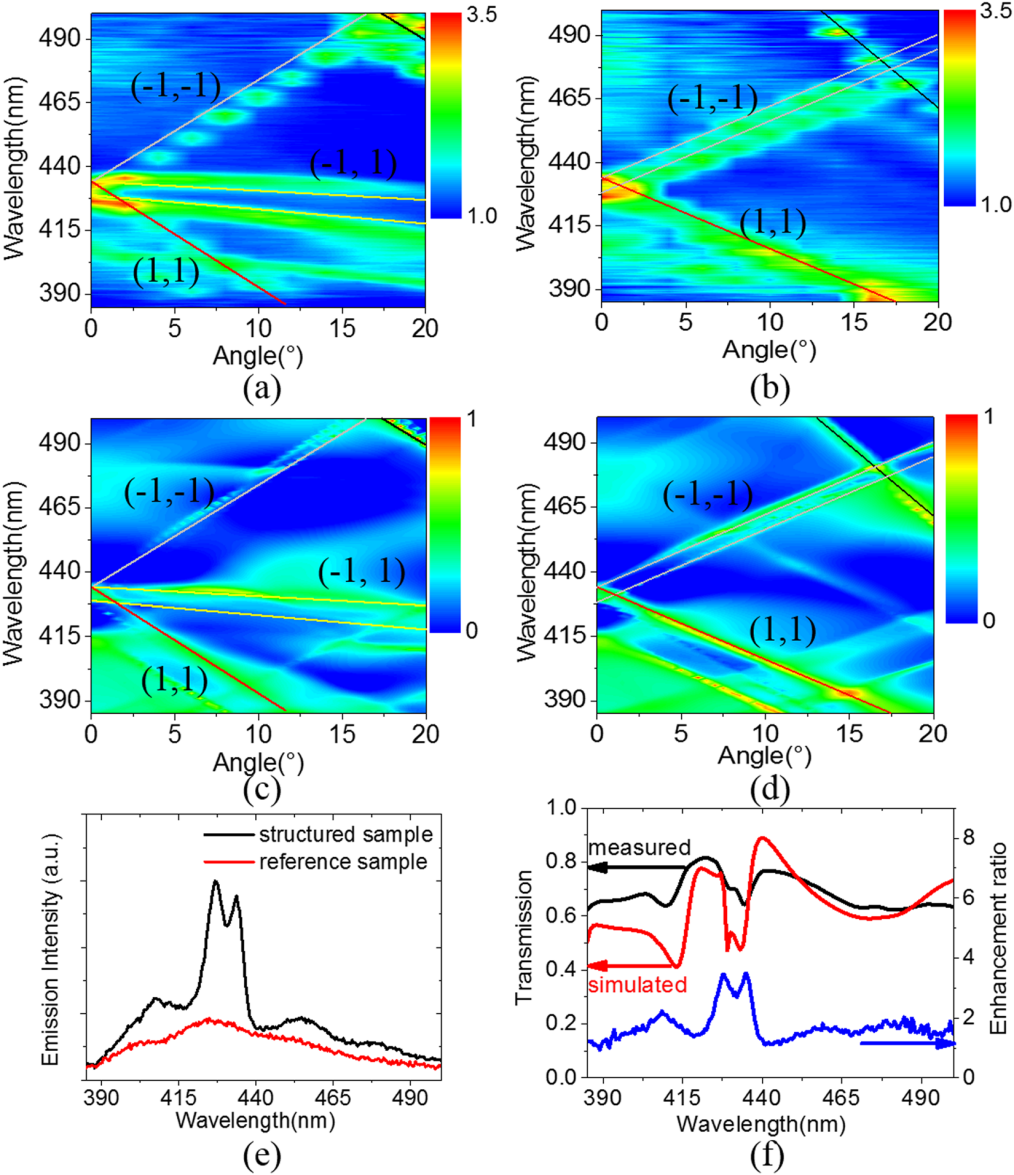
Measured angle-dependent emission enhancement spectra along ΓM **(a)** and ΓX **(b)** orientations. Simulated angle-dependent extinction spectra along ΓM **(c)** and ΓX **(d)** orientations. **(e)** Emission spectra of the structured sample and the reference sample in the normal direction. **(f)** Measured emission enhancement spectrum, measured zero-order transmission spectrum, and simulated zero-order transmission spectrum in the normal direction.

Figure 4(a,b) show the measured angle-dependent emission enhancement spectra along ΓM and ΓX orientations, respectively. The emission enhancement spectra are defined as the ratio of the emission spectra of the structured sample to the reference sample. It is found that the enhancement spectra exhibit distinctive dispersion bands. The spectra in the range of 420 to 440 nm are significantly enhanced in the normal direction, which are well matched with the luminescence peak of the plastic luminescent film. Figure 4(c,d) show the simulated angle-dependent extinction spectra along ΓM and ΓX orientations, respectively.

The simulated extinction spectra are fundamentally matched with the characteristic of measured emission enhancement spectra, indicating that the diffracted modes create new pathways for the emitting light to escape into free space. The corresponding diffracted orders are plotted with different colored curves, assuming that the structures were embedded in a homogeneous refractive index medium. The diffracted modes along the ΓM and ΓX orientations satisfy the Equations (1) and (2) respectively.

1 $${(\frac{\sqrt{2}}{2}{k}_{0}\sin \theta +{m}_{1}\frac{2\pi }{a})}^{2}+{(\frac{\sqrt{2}}{2}{k}_{0}\cos \theta +{m}_{2}\frac{2\pi }{a})}^{2}={{k}_{1}}^{2}$$

2 $${({k}_{0}\sin \theta +{m}_{1}\frac{2\pi }{a})}^{2}+{({m}_{2}\frac{2\pi }{a})}^{2}={{k}_{1}}^{2}$$

where *k*_0_ is the incident wave number in vacuum, *θ* is the incident angle, *k*_1_ is the diffractive wave number, *a* is the lattice constant of the photonic crystals, and *m*_1_ and *m*_2_ are integers defining the diffractive order.

The effective refractive indices of 1.52 and 1.53 are corresponding to the peak wavelengths of 429 nm and 433 nm, respectively, in the extinction spectra along the normal direction. Figure 4(e) shows the emission spectra of the structured sample and the reference sample in the normal direction. The emission spectrum of the structured sample exhibits the enhancement in the entire wavelength range and the enhancement ratio is maximum at the luminescence peak. For a clear comparison, the simulated zero-order transmission spectrum, the measured transmission spectrum, and the enhancement spectrum in the normal direction were plotted in Fig. 4(f). It is found that a uniform profile with two apparent adjacent dips in the range of 420–440 nm for the transmission spectra. The dips of the zero-order transmission spectrum are consistent with the peaks of the emission enhancement spectrum, indicating that the emission enhancement is attributed to the coupling of the luminescence center with the photonic crystal modes^30^. One can find that there are some differences between the simulations and the experiment data because the simulated structures are not exactly the same as the experiments. First, the simulated structures are perfectly periodic, while there are some defects in the prepared structures due to the stitching errors. Second, in order to simplify the simulation, the refractive index of the TiO_2_ used in the simulation was set as a constant value of 2.8. However, such differences cannot prevent us from interpreting the experimental phenomena and understanding the related physics.

**Figure 5:**
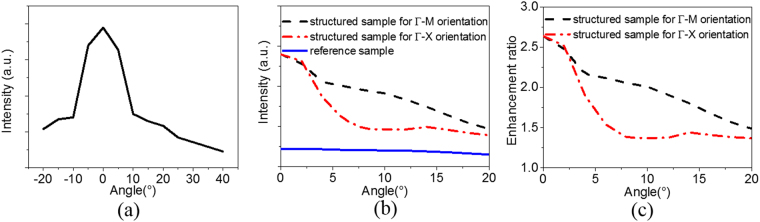
**(a)** Wavelength-integrated emission intensities in the range of 385–500 nm as a function of the excitation angle, fixing the emission angle at 15° along ΓM orientation. **(b)** Angle-dependent wavelength-integrated emission spectra in the range of 420–440 nm for the structured sample in the ΓM and ΓX orientations and the reference sample. **(c)** The enhancement ratio of the structured sample relative to the reference sample.

In order to observe the enhancement of photonic crystals attributed from ultra-violet excitation source, the wavelength-integrated emission intensities in the range of 385–500 nm were measured, monitoring the emission angle at 15° along ΓM orientation, as shown in Fig. 5(a). It is found that the photonic crystal structures can obtain the excitation enhancement in the normal direction, but cannot lead to the excitation enhancement at 25° along ΓM orientation. Therefore, selecting the excitation angle at 25° along ΓM orientation is reasonable in that the factor of excitation enhancement can be efficiently excluded. Angle-dependent wavelength-integrated emission spectra in the range of 420–440 nm for the structured sample in the ΓM and ΓX orientations and the reference sample were presented in Fig. 5(b). The emission of the structured sample is stronger than that of the reference sample in the whole angle range. Figure 5(c) presents the enhancement ratio of the structured sample relative to the reference sample. In the normal direction, the wavelength-integrated emission intensity of the structured sample is 2.64-folds enhancement relative to the reference sample, which is similar to the result of near 3-fold enhancement for a upconversion fluorescence film of NaYF_4_:Yb^3+^,Tm^3+^ nanocrystal^42^. The enhancement effect could be beneficial for the light collection for the application in detection systems.

**Figure 6:**
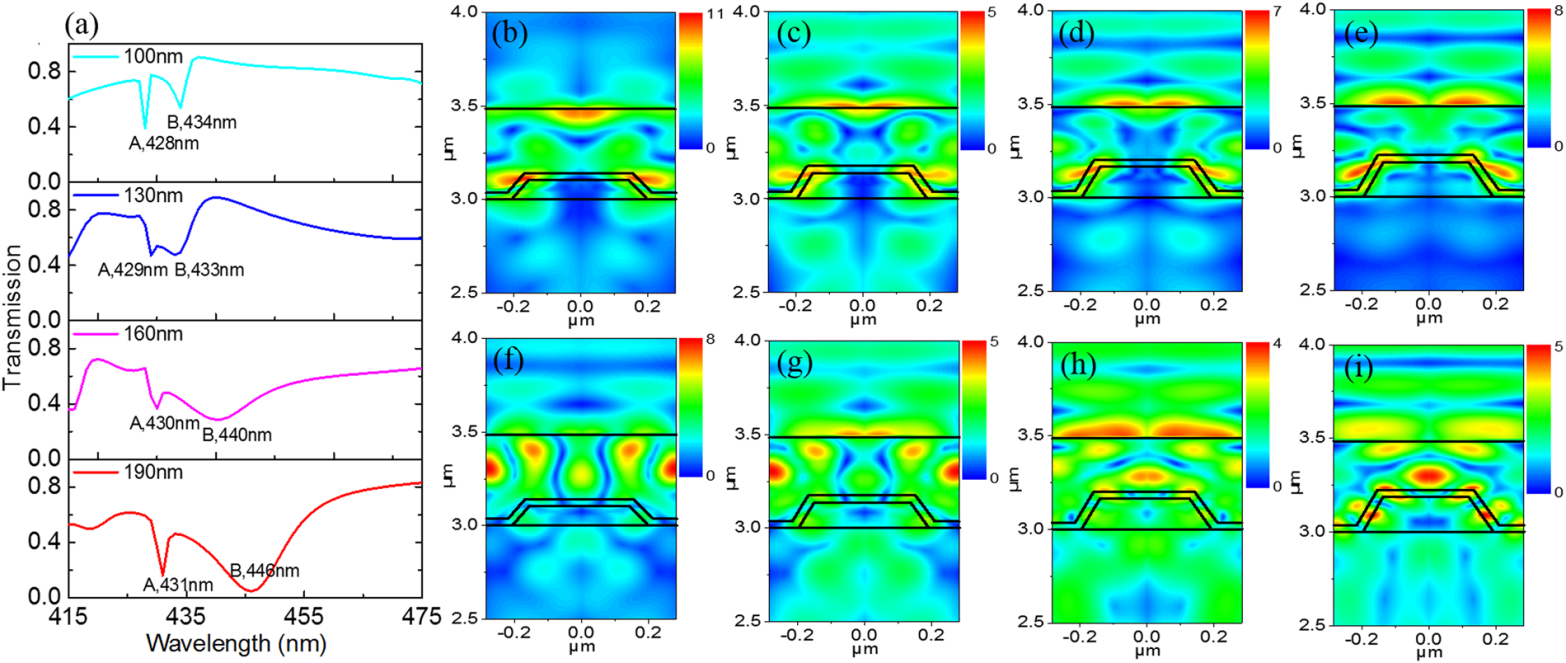
**(a)** Simulated transmission spectra in the normal direction with different height of nanopillars from 100 to 190 nm. Simulated spatial distribution of electric-field intensity with the pillar height and the wavelength of h = 100 nm and λ = 428 nm **(b)**, h = 130 nm and λ = 429 nm **(c)**, h = 160 nm and λ = 430 nm **(d)**, h = 190 nm and λ = 431 nm **(e)**, h = 100 nm and λ = 434 nm **(f)**, h = 130 nm and λ = 433 nm **(g)**, h = 160 nm and λ = 440 nm **(h)**, h = 190 nm and λ = 446 nm **(i)**.

In order to understand the influence of the height of the individual nanopillars on the emission control, the simulated zero-order transmission spectra with the height of nanopillars ranging from 100 to 190 nm are shown in Fig. 6(a). It is found that there are two dips (labeled as Dip A and Dip B) responsible for the diffracted modes with different characters. Dip A exhibits narrow band character and slowly redshifts from 428 to 431 nm as the height of the nanopillars increases. Dip A originates from the guided-mode resonance which is strongly controlled by the period and the aspect ratio of the photonic crystals. In contrast, as the height of the nanopillars increases, Dip B becomes much broader and has a significant redshift from 434 to 446 nm. Such character suggests that Dip B is produced by the Fabry-Perot resonance^22^. To further confirm this judgment, the simulated spatial distributions of the electric-field intensity are shown in Fig. 6(b–i). It is apparent from Fig. 6(b–e) that for the case of Dip A, the electric-field intensity is mainly localized in the photonic crystals. While for the case of Dip B shown in Fig. 6(f–i), the electric-field intensity is mainly localized in the luminescent layer. This is a typical Fabry-Perot resonance character in that the distance of the above and the below surfaces strongly affects the resonance wavelength. As a result, the height of nanopillars can be designed to control the optical properties of photonic crystals.

In conclusion, photonic crystals with a large height of individual nanopillars for a large area have been fabricated by the method based on the combination of XIL and RIE. Such photonic crystals can be used to control the directionality of emission from a plastic luminescent film. The experimental result shows a wavelength-integrated enhancement of 2.64-folds in the range of 420 to 440 nm in the normal direction. Simulations reveal that the directional emission enhancement is attributed to the guided-mode resonance and Fabry-Perot resonance. The present demonstration could be beneficial to the applications where directional emission is required.

## Methods

### Photonic crystals fabrication

Firstly, the poly-methyl-methacrylate (PMMA, 950 PMMA A2, MicroChem Corp., 2% solids) photoresist was spin-coated onto a cleaned quartz substrate with a speed of 2000 rpm for 50 s, and then baked at 180 °C for 90 s. The thickness of the prepared PMMA (n = 1.5) film was about 80 nm. Subsequently, on the surface of PMMA film, a layer of hydrogen silsesquioxane (HSQ, XR 1541, Dow Corning, 4% solids) with a thickness of 50 nm was spin-coated at 5000 rpm for 45 s and baked at 150 °C for 180 s. The patterns on HSQ (n = 1.4) layer were fabricated by XIL beamline (BL08U1B) at Shanghai Synchrotron Radiation Facility (SSRF)^38^. The soft X-ray with energy of 92.5 eV generated by elliptically polarized undulator (EPU) was used to exposure the HSQ photoresist. In the process of XIL, the exposure dose is 620 mJ/cm^2^ and a mask consisting of four diffraction gratings was utilized to form interfering beams. An area of 0.4 × 0.4 mm^2^ was obtained after an individual exposure. Taking advantage of the shutter control system and the step motor control system, the structured area with the size of 5.2 × 5.2 mm^2^ was accomplished. After development in tetramethyl ammonium hydroxide (TMAH, 25% solution in water), an array of conical HSQ nanopillars was obtained. After that, using the HSQ nanopillars as the etching mask, the underlying PMMA layer was etched with oxygen plasma by the dry etcher (OXFORD INSTRUMENTS, Plasmalab System 100). Given the RIE condition of 200 W radio frequency power, 40 mTorr pressure and 45 sccm oxygen flow rate, the etch rate was 10 nm/min.

### Conformal layer deposition

The conformal layer of TiO_2_ was deposited on the surface of the nanopillars using an atomic layer deposition (ALD) system (Picsun SUNALE R-200). During the deposition, TiCl_4_ and H_2_O were used as the precursors of Ti and O, respectively. The deposition temperature and pressure in the reaction chamber were kept at 75 °C and 17 hPa. The thickness of conformal layer was controlled by the numbers of the ALD cycles. In each ALD reaction cycle, the reaction chamber was replenished with precursors by a 0.3 s pulse and then purged with N_2_ for 18 s. After 500 cycles, the thickness of about 40 nm was obtained.

### Spin-coating luminescent film

The luminescent material doped with p-terphenyl (4% by weight) and POPOP (1.4-bis-(5-phenyl-2-oxazolyl), 0.02% by weight) was fully dissolved in toluene. Then the prepared solution was pipetted onto the surface of the prepared photonic crystals and spun at 4000 rpm for 120 s. After laying up for 48 hours, the luminescent film (n = 1.59) was solidified and the thickness is about 450 nm.

### Spectra measurements

An ultraviolet light emitting diode with the emitting wavelength of 365 nm was used as the excitation source and a fiber spectrometer with a FFT-CCD (PG2000-Pro-EX, Ideaoptics Co.) was applied to measure the emission spectra. The excitation angle deviating from the normal direction was set as 25° along ΓM orientation and the emission emergence angle was relative to the normal direction. In order to measure angle-dependent emission spectra, the excitation source and the sample were fixed at the edge and the center, respectively, of a circular platform which could rotate and display the rotation angle. The fiber spectrometer was fixed far away from the platform.

### Simulations

The simulation is based on rigorous coupled wave analysis with the codes from RSoft Design Group. The extinction spectrum is defined as 1 − *T*_0_, where *T*_0_ is the zero-order transmission spectrum. During the simulations, a plane wave with averaged s- and p-polarization incidents to the structured sample, and the Bloch boundary condition was applied. All materials were assumed to be lossless and constant refractive indices. The harmonics was set as 6 in horizontal direction.
